# IL1RAP-expressing myeloid-stromal networks represent a therapeutic vulnerability to improve chemoimmunotherapy sensitivity in pancreatic cancer

**DOI:** 10.1172/jci.insight.202487

**Published:** 2026-06-22

**Authors:** Erin M. Dickey, Harper M. Marsh, Camilla Rydberg-Millrud, Haleh Amirian, Karthik Rajkumar, Manan Patel, Andrew Adams, Anuroop Allena, Kevin Van der Jeught, Nipun Merchant, Peter J. Hosein, Anna Bianchi, David Liberg, Jashodeep Datta

**Affiliations:** 1 Department of Surgery and Sylvester Comprehensive Cancer Center, University of Miami Miller School of Medicine, Miami, Florida, USA.; 2 Cantargia AB, Lund, Sweden.; 3 Department of Microbiology/Immunology and; 4 Department of Medicine, University of Miami Miller School of Medicine, Miami, Florida, USA.

**Keywords:** Immunology, Oncology, Cancer immunotherapy, Drug therapy, Innate immunity

## Abstract

IL1RAP blockade remodels the pancreatic cancer microenvironment, reduces myeloid immunosuppression, restores memory-like CD8+ T cell states, and enhances chemoimmunotherapy sensitivity.

**To the Editor:** Pancreatic ductal adenocarcinoma (PDAC) remains refractory to chemotherapy and immunotherapy due to a tumor microenvironment (TME) characterized by stromal inflammation and myeloid-derived immunosuppression (1). Among the molecular drivers of chemoimmunoresistance is IL-1 family cytokine (IL-1α/β,IL-33,IL-36) signaling, which converges on the IL-1 Receptor Accessory Protein (IL1RAP) coreceptor for downstream immunomodulatory effects (2), reinforcing proinflammatory circuitry in the TME. High IL1RAP expression correlates with poor survival in PDAC (Supplemental Figure 1A; supplemental material available online with this article; https://doi.org/10.1172/jci.insight.202487DS1), nominating it as a prognostic biomarker and therapeutic vulnerability.

To interrogate IL1RAP as a driver of therapeutic resistance, we integrated post hoc trial transcriptomic analyses with scRNA-seq and biomarker data. In the COMPASS trial (3), chemotherapy-resistant (progressive disease; *n* = 39) advanced PDAC tumor transcriptomes exhibited enrichment of IL1RAP-related pathways versus chemotherapy-responsive (partial response + stable disease; *n* = 156; Supplemental Figure 1B). Complementary analyses of Human Tumor Atlas Network (HTAN) PDAC scRNA-seq atlas (4) showed elevated *IL1RAP* expression across immune/myeloid, stromal/cancer-associated fibroblast (CAF), and tumor/acinar cell compartments (Figure 1A and Supplemental Figure 1C). Notably, *IL1RAP* was enriched in chemotherapy-resistant samples not only in tumor-cell but also immune/myeloid and stromal/CAF transcriptomes (Figure 1A).

We next analyzed post hoc data from the CANFOUR trial (NCT03267316), evaluating anti-IL1RAP antibody nadunolimab plus gemcitabine/nab-paclitaxel (GnP) in advanced PDAC (5). Patients with high baseline tumor cell–IL1RAP expression had superior overall survival following nadunolimab + GnP treatment (5). Extending HTAN-based findings, stratification by stromal and immune IL1RAP expression (Supplemental Figure 1D) revealed that elevated stromal/CAF (*n* = 34; *P* = 0.0058) and immune (*n* = 19; *P* = 0.014) IL1RAP expression each associated with prolonged duration of response to nadunolimab + GnP (Figure 1B), suggesting IL1RAP-expressing immune-stromal compartments as a therapeutic barrier in PDAC. We therefore hypothesized that IL1RAP inhibition — by mitigating myeloid-CAF networks and T cell exclusion/dysfunction — may sensitize PDAC to cytotoxic and immunomodulatory therapies.

To test this hypothesis in vivo, we employed the autochthonous *Ptf1a^Cre/+^;LSL-Kras^G12D/+^;Tgfbr2^fl/fl^* (PKT) model, which phenocopies the stromatogenic and inflammatory TME of human PDAC (6), particularly robust *Il1rap* gene module expression across tumor/epithelial, stromal/CAF, and immune/myeloid compartments by scRNA-seq (Figure 1C) — mirroring HTAN findings. Treatment of tumor-bearing PKT mice with murine analogs of clinical-grade nadunolimab (mNadu; 10 mg/kg 3×/week) (2) significantly restrained tumor growth (*P* = 0.019; Figure 1D and Supplemental Figure 1E) and CK19^+^ epithelial proliferation (*P* = 0.002; Supplemental Figure 1F) versus isotype. Histology showed reduced stromal fibrosis (Sirius red; *P* = 0.0003) and desmoplasia (Masson’s trichrome stain; *P* = 0.0088) in mNadu-treated tumors (Supplemental Figure 1G), underscoring the effect of IL1RAP blockade on TME architecture.

To define the stromal-immune TME remodeling underlying tumor regression after IL1RAP inhibition in PKT mice, we profiled treated murine and human tumors across orthogonal analytic platforms. mNadu treatment reduced intratumoral CD11b^+^ myeloid cells (*P* = 0.0077), while increasing CD3^+^ T cell infiltration (*P* = 0.0033) via flow cytometry (FACS; Figure 1E). In paired human PDAC biopsies obtained pre-/postnadunolimab ± GnP treatment (*n* = 2 each), immunofluorescence showed reduced CD11B^+^CD14^+^ and/or CD11B^+^CD15^+^ myeloid cells and increased intratumoral Granzyme B/Ki67^+^ CD8^+^ T cells within tumor nests following nadunolimab treatment (Figure 1F and Supplemental Figure 2A). Cytometric analysis of CD8^+^ T cell subsets in mNadu-treated tumors revealed specific expansion of Ly108^+^CD69^+/–^ memory progenitor-exhausted T cells (*P* = 0.0278) and reduction in terminally exhausted Ly108^–^CD69^+^ T cells (*P* = 0.0084; Figure 1G).

To resolve cell-specific effects of IL1RAP blockade, we performed scRNA-seq on mNadu- and isotype-treated PKT tumors (Figure 1H). IL1RAP inhibition redistributed single-cell compartments, with contraction of myeloid cell clusters and modest expansion of T cell clusters (Supplemental Figure 2B). To assess functional consequences of this immune-permissive TME remodeling, scRNA-seq profiling showed transcriptional reprogramming of myeloid cells in mNadu-treated tumors, with downregulation of immunosuppressive programs (*Arg1*, *Cybb*, *Chil3*, *Cd177*, *Camp*, IL-1/TNF signaling) and upregulation of type I IFN responses and antigen presentation/processing pathways (Figure 1I and Supplemental Figure 2C). In parallel, T cell transcriptomes skewed toward memory/stem-like reprogramming marked by TCR engagement, IFN-γ response, and IL-2–driven CD8^+^ activation, with mitigation of dysfunctional/regulatory signatures (Figure 1I). This reprogramming was corroborated by immunofluorescence, with reduction in CD206^+^F4/80^+^ M2-like macrophages and enrichment in MHC-II^+^F4/80^+^ M1-like macrophages and CD3^+^ T cells in mNadu-treated tumors (all *P* < 0.05; Supplemental Figure 2D). These data suggest that disrupting IL1RAP myeloid-stromal networks may induce T cell pools permissive to checkpoint blockade.

To test this, we treated PKT mice with mNadu combined with gemcitabine/paclitaxel (GP) and anti-PD1. The 4-drug regimen significantly extended survival compared with isotype alone or chemoimmunotherapy(GP + anti-PD1) alone (median 54d vs. 43d vs. 43d, *P* < 0.001). Notably, mNadu monotherapy also improved survival over isotype treatment (median 50d vs. 43d, *P* = 0.023; Figure 1J).

By integrating post hoc clinical trial analysis, single-cell profiling of human PDAC, and mechanistic interrogation in preclinical models, we identify IL1RAP-expressing myeloid-stromal TME networks as an actionable therapeutic barrier in PDAC. Expanding on prior findings that targeting IL1RAP^+^ CAFs restrains myeloid-enriched TMEs (2), we demonstrate that pharmacologic disruption of IL1RAP-dependent myeloid–stromal networks reprograms inflammatory signaling and restores memory/stem-like T cells to improve chemoimmunotherapy sensitivity.

Our data nominate IL1RAP as a therapeutic vulnerability and predictive biomarker for combination strategies in PDAC. Accordingly, a neoadjuvant trial testing nadunolimab + chemoimmunotherapy in patients with operable PDAC is near-deployment, offering a path to unlocking immunotherapy responsiveness in a malignancy considered immunologically inert.

## Conflict of interest

JD receives funding from Cantargia AB and consultant fees from Boston Scientific.

## Funding support

This work is the result of NIH funding, in whole or in part, and is subject to the NIH Public Access Policy. Through acceptance of this federal funding, the NIH has been given a right to make the work publicly available in PubMed Central.

- Research reported in this publication was supported by the National Cancer Institute of the NIH under Award #P30CA240139
- EMD was supported by the NIH under a training grant T32CA211023.
- This work was supported by a research contract from Cantargia AB
- This work was supported by the U.S. Department of Defense Idea Development Award grant #HT9425310699

## Supplementary Material

Supplemental data

Supporting data values

## Figures and Tables

**Figure 1:**
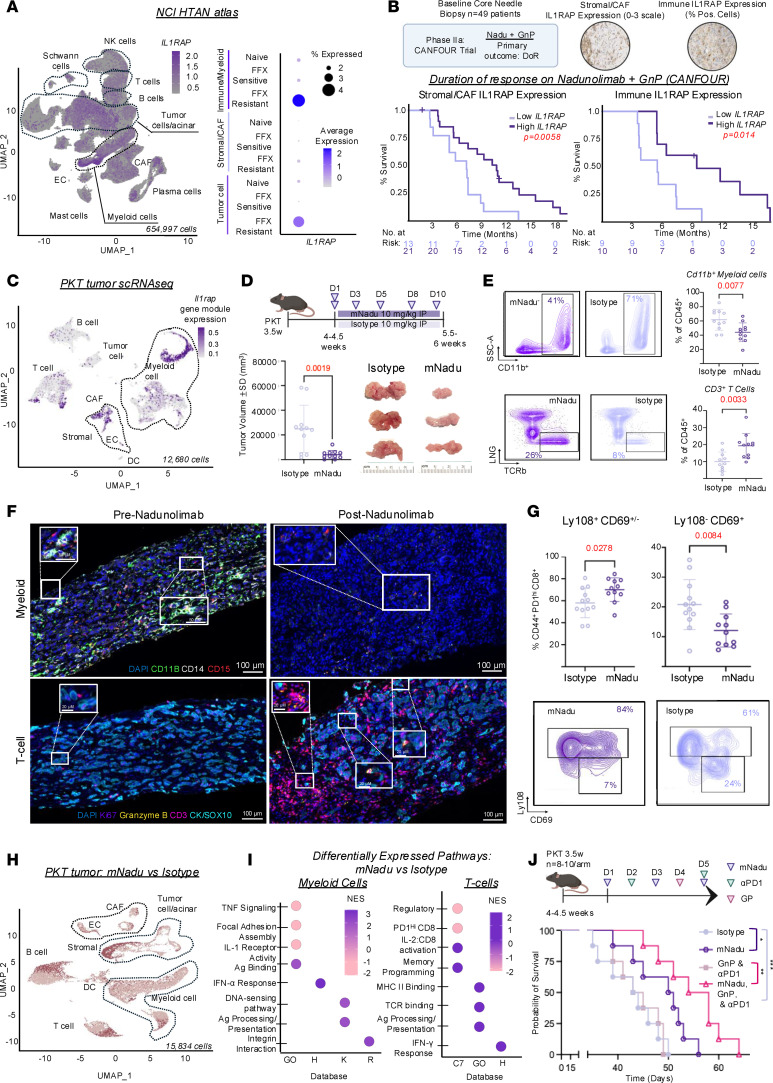
IL1RAP-expressing myeloid-stromal networks are a therapeutic barrier in PDAC. (**A**) UMAP of 654,997 cells from HTAN PDAC dataset (*n* = 79); *IL1RAP* expression across immune, stromal, and tumor-cell compartments in naive (*n* = 7), FOLFIRINOX-sensitive (FFX-sensitive) (*n* = 5), and resistant (*n* = 3) subgroups. (**B**) CANFOUR trial: nadunolimab (Nadu) + gemcitabine/nab-paclitaxel (GnP) in metastatic PDAC; stromal/CAF and immune IL1RAP quantification from tumor biopsies (*n* = 49; top). Duration of response to nadunolimab + GnP stratified by stromal (*n* = 24, HR: 0.41;95%CI, 0.18–0.94) or immune (*n* = 19, HR: 0.36; 95%CI, 0.13–1.02) IL1RAP IHC (2-tailed log-rank test, right). (**C**) UMAP of scRNA-seq clusters from PKT tumors showing IL1RAP gene module (*Il1rap/Il1r1/Il1rl2/Il1rl1*). (**D**) PKT mice treated with mNadu or isotype; tumor volumes shown (*n* = 11/group). (**E**) FACS quantification of CD11b^+^ myeloid and CD3^+^ T cell frequencies (%CD45^+^) in PKT tumors (*n* = 11/group, 2-tailed *t* test). (**F**) Immunofluorescence of paired PDAC biopsies pre/postnadunolimab ± GnP. Scale bar: 100 μm. (**G**) FACS of CD8^+^PD1^hi^CD44^+^ T cells by Ly108/CD69 status, indicating memory progenitor-exhausted and terminally exhausted (*n* = 11/group, 2-tailed *t* test). (**H** and **I**) UMAP of 15,834 scRNA-seq profiles and pathway enrichment in myeloid (left) and T cell (right) single-cell clusters, from isotype vs. mNadu-treated PKT tumors (*n* = 3/group; FDR *q* < 0.05). (**J**) Schema and survival of PKT mice treated with isotype, mNadu, GP + anti-PD1, or mNadu + GP + anti-PD1 (*n* = 8/group).* *P* < 0.05, **P= < 0.01, ***P < 0. 001.

## Version 1 06/22/2026

Electronic publication
