# Supplementary material for: IL1RAP-expressing myeloid-stromal networks represent a therapeutic vulnerability to improve chemoimmunotherapy sensitivity in pancreatic cancer
Source: JCI Insight. 2026 Jun 22;11(12):e202487. doi: 10.1172/jci.insight.202487 (PMC13313497; doi:10.1172/jci.insight.202487)
Supplement: Supplemental data [file jciinsight-11-202487-s046.pdf]

SUPPLEMENTARY MATERIALS AND METHODS

Sex as a biological variable: Equal numbers of male and female mice were used in survival and endpoint analyses to control for potential sex-related bias.

***Bulk RNA-seq analysis*:** Differentially expressed genes (ranked gene lists with effect sizes)—but not sample-level expression matrices—and corresponding survival annotations from the COMPASS clinical trial were accessed through a collaboration with the Ontario Institute for Cancer Research, University Health Network, Toronto, Canada (1). Gene symbols were converted to Entrez gene identifiers using org.Hs.eg.db (v3.20.0) via clusterProfiler (v4.16.0). For Gene Set Enrichment Analysis (GSEA) (**Figure S1A**), genes were ranked by decreasing log fold-change to generate a pre-ranked list for GSEA. Enrichment testing was conducted using clusterProfiler and ReactomePA (v1.52.0), applying 10,000 permutations and controlling false discovery with the Benjamini–Hochberg procedure. Pathways meeting an adjusted p value threshold of < 0.05 were retained for interpretation and plotting. Visualization was performed in ggplot2 (v3.5.2). To contextualize transcriptomic findings within broader clinical cohorts, survival associations were additionally evaluated using The Cancer Genome Atlas (TCGA) through the GEPIA platform. Overall survival and disease-free survival were assessed by stratifying patients into high- versus low-expression groups for IL1RAP, using the cohort median as the cutoff.

Single-cell RNA-seq processing and pathway analysis
Human pancreatic tumor scRNA-seq (HTAN): Single-cell RNA-sequencing datasets from human pancreatic tumors were obtained from the Human Tumor Atlas Network (HTAN) (2), derived from the Washington University Human Tumor Atlas Research Center. Data were processed locally in R (v5.1.0) using Seurat (v5.1.0). Raw 10x count matrices were imported per sample using Read10X and converted into sample-specific Seurat objects with thresholds of min.cells = 3 and min.features = 200. Sample objects were then merged. Samples exhibiting inconsistent assay formatting or missing RNA assays were excluded. Cells with >10% mitochondrial transcript content were excluded. Normalization and variance stabilization were performed using SCTransform. Dimensionality reduction was performed via Principal Component Analysis (PCA) on highly variable genes, retaining the subset of PCs explaining >90% of variance. Batch correction was conducted using Harmony integration and UMAP embeddings were computed using Harmony-corrected PCs, followed by neighbor graph construction and unsupervised clustering (resolution = 0.1-0.5). Clusters were annotated based on canonical lineage-specific gene expression.
PKT scRNA-seq: ScRNAseq of treatment-naïve tumors from 6.5-week-old *Ptf1a*$^{Cre/+}$;*LSL-Kras*$^{G12D/+}$;*Tgfbr2*$^{fl/fl}$ (PKT) mice (pooled from n=3 mice) and used in analysis **Figure 1C** was previously reported (3). Pancreatic tumors from PKT mice treated with either mNadu or isotype vehicle control were subjected to scRNAseq as described previously (3). Briefly, the 10x Genomics Chromium Single Cell 3’ Reagent v3.1(Cat # PN-1000268) was used for 3’ transcriptional profiling where cell suspensions were processed with standard conditions and volumes. Single cell suspensions from

tumors derived from PKT mice were isolated, and live cells were sorted using flow cytometry. Volumes were designed for a target cell recovery of 100,000 cells and initialized on the Chromium Controller per manufacturer's guidelines. The resultant purified cDNAs were quantified and assessed on the Agilent Bioanalyzer using the High Sensitivity DNA Kit (Cat #5067-4626). The final single cell 3' library was quantified using the Qubit dsDNA High Sensitivity (Cat #Q33231) and evaluated on the Agilent Bioanalyzer using the High Sensitivity DNA Kit. For sequencing, libraries were loaded at optimized concentrations onto an Illumina NovaSeq and paired end sequenced under recommended settings (R1: 28 cycles; i7 index: 10 cycles; i5 index: 10 cycles; R2: 90 cycles). The libraries were diluted to varying nM concentrations in Illumina Resuspension Buffer (PN-15026770), and denatured according to Illumina standard guidelines, then loaded on the Illumina NovaSeq at 1.2 nanomolar. The resulting intensity files were demultiplexed as FASTQ files using Illumina BaseSpace software which were aligned to the transcriptome using the 10x Genomics CellRanger (ver4.0.0) software package. Gene symbols were converted to Entrez identifiers using org.Mm.eg.db (v3.21.0) via clusterProfiler (v4.16.0). Marker gene lists derived from myeloid and T-cell clusters were analyzed separately.

Single-cell pathway enrichment: Gene symbols were converted to Entrez gene identifiers using org.Mm.eg.db (version 3.21.0). Ranked gene lists were generated by ordering genes in decreasing log fold-change. GSEA was performed against KEGG, Reactome, Gene Ontology Biological Process and Molecular Function terms, and gene sets obtained from the Molecular Signatures Database (MSigDB) using the msigdbr package (version 7.5.1). Enrichment testing was conducted using clusterProfiler and ReactomePA (v1.52.0), applying 10,000 permutations and controlling false discovery with the Benjamini–Hochberg procedure. Pathways meeting an adjusted *p* value threshold of < 0.05 were retained for interpretation and plotting. Visualization was performed in ggplot2 (v3.5.2).

In vivo studies*:* PKT mice were generated as previously described (3). Briefly, *Ptf1a* $^{Cre/+}$; *Tgfbr2* $^{flox/flox}$ and *LSL-Kras* $^{G12D/+}$; *Tgfbr2* $^{flox/flox}$ mice were originally supplied by Dr. Hal Moses (Vanderbilt University, Nashville, TN). These intercrosses generated *Ptf1a*$^{Cre/+}$;*LSLKras*$^{G12D/+}$;*Tgfbr2*$^{flox/flox}$ mice (PKT) mice on a C57Bl/6 background. Genotyping was performed using established oligonucleotide primer sets to confirm the presence of the targeted alleles. For endpoint analyses (e.g., flow cytometry, histology), PKT mice received intraperitoneal injections of mNadu (Cantargia AB; anti-mouse surrogate of human IgG1 IL1RAP inhibitor nadunolimab; 10 mg/kg[7]) or isotype control, twice on the first day of treatment (at 4-4.5 weeks of age), followed by 3x weekly dosing for two weeks, after which mice were sacrificed. For survival analysis, combinations of mNadu (10mg/kg, regimen as described above), gemcitabine (100mg/kg, i.p. weekly), paclitaxel (10mg/kg, i.p. weekly), and anti-PD1 antibody (BioXCell, Clone 29F.1A12, 200μg/mouse, i.p. 2x weekly) were given. Treatments were initiated at 4 weeks of age and continued until mice were euthanized due to severe morbidity or when tumor burden exceeded 10% of body weight. Tumor volumes were monitored by ultrasound as previously described (3).

Tissue processing: Tissue processing for endpoint analysis was performed as previously described (3). Briefly, spleens were harvested from PKT mice treated with either mNadu or isotype control and passaged processed in RBC lysis buffer, then passaged through a 100 µm mesh strainer to generate single-cell suspensions of splenocytes. Whole pancreata from the same cohort of PKT mice (mNadu- or isotype treated) were digested in RPMI containing 0.6 mg/mL collagenase P (Sigma-Aldrich, #11213857001), 0.8 mg/mL collagenase V (Sigma-Aldrich, #9001-12-1), 0.6 mg/mL soybean trypsin inhibitor (Sigma-Aldrich, #9035-81-8), and 1800 U/mL DNase I (ThermoFisher Scientific, #18047019) for 20-30 minutes at 37 °C. Samples were subsequently washed in cold PBS and passaged through 40 µm mesh strainers to obtain single-cell suspensions. Cell suspensions were used for downstream analyses or cryopreserved at -80 °C.

IHC analysis and staining: Tumor biopsies obtained at study screening or archival biopsies from patients enrolled in the CANFOUR trial (4) were utilized to assess tumor expression of IL1RAP (polyclonal rbIgG; Cantargia AB). Samples were formalin-fixed, paraffin-embedded, and sectioned at 3 µm, treated with Tris-EDTA antigen retrieval (pH 9), and stained with antibodies targeting IL1RAP (7 µg/ml; Cantargia) on a Leica Bond III automaton. All sections were counterstained with hematoxylin and Casein-diluent. In the stromal/CAF compartment, IL1RAP expression was scored on a 0-3 scale, and in the immune compartment it was quantified as the percentage of IL1RAP$^+$ cells. All morphological assessments were performed by board-certified pathologist Dr. Pierre Lefesvre (Universitair Ziekenhuis Brussel), using established histopathologic criteria. Patients were subsequently stratified into “low” or “high” IL1RAP expression groups, defined either by a median cut-point (IL1RAP stroma) or by determination of the optimal cut-point (IL1RAP immune cells) using the *survminer* package in R. For Kaplan-Meier analyses, the median follow-up for each group was as follows: IL1RAP stromal analysis—14.4 months for all patients, 18.9 months for IL1RAP-high (n=21), and 10.9 months for IL1RAP-low (n=13); IL1RAP immune cell analysis—20.0 months for all patients, 27.5 months for IL1RAP-high (n=10), and 17.7 months for IL1RAP-low (n=9).

Histological analysis from PKT mice treated with isotype or mNadu was performed as described previously (3). Tissues were fixed in 10% neutral-buffered formalin, transferred to 70% ethanol solution, and paraffin-embedded. Hematoxylin and eosin (H&E), Sirius Red, Masson’s Trichrome, Alcian Blue, and cytokeratin-19 (CK19) immunohistochemistry was performed by HistoWiz Inc. For quantitative analysis of special stains samples in **Figure S1F-G**, ImageJ image analysis software (NIH, Bethesda MD) was utilized for quantification of positive staining and reported as percentage area of positive staining. The analyzing researcher was blinded to the sample identities before analysis. For immunofluorescence images in **Figure S2D**, antigen retrieval was performed using microwaved 1X Citrate Buffer followed by heating at sub-boiling temperature for 15 min. The slides were later blocked using BlockAid buffer for 1 h and probed using primary antibodies anti mouse CD3e (Thermo Fisher Scientific #14-0033-82), F4/80 (Thermo Fisher Scientific **#** MA5-16363), MHC-II (Thermo Fisher Scientific **#**14-5321-82), CD206 (Thermo Fisher Scientific **#** PA5-46994)

overnight. The slides were washed and probed with fluorophore tagged secondary antibody for 1h and counterstained with DAPI. The tissue sections were then imaged on Andor Dragonfly spinning Disk confocal and Olympus VS120. The images were analyzed with Imaris Viewer v.11.0.0. The images were analyzed using QuPath (v0.4+). Regions of interest were manually annotated, and cell detection was performed on the DAPI channel using the built-in cell detection algorithm (background radius 8 μm, sigma 2 μm, cell expansion 6 μm, size filter 35-400 μm²). Cells were classified for individual markers using single-measurement classifiers thresholded on mean cytoplasmic fluorescence intensity, with thresholds set empirically per marker. Composite classifiers were used to identify co-expressing cell populations. All classifiers were applied in a single unified pipeline to prevent sequential overwriting of cell classifications. Cell counts for each population were normalized to total cell count per ROI and exported for statistical analysis.

Multiplexed immunofluorescence (mIF) of human PDAC biopsies: Available pre- *and* post-treatment biopsies from patients receiving either nadunolimab monotherapy (n=1) or nadunolimab + gemcitabine/nab-paclitaxel (n=1) were sectioned with a thickness of 4μm at Cerba Research Montpellier (Montpellier, France) and mounted on the same slide. Multiplex staining was performed using Ultivue InSituPlex® panels: MDSC FixVUE (CD11b clone SP331, CD14 clone SP192, and CD15 clone Leu-M1), and T-act FixVUE (Ki67 clone SP6, Granzyme B clone EPR8260, CD3 clone BC33, PanCK/SOX10 clone AE1/AE3_BC34). H&E staining was performed in parallel. mIF was quantified as percentage of positive cells and as cell density (cells/mm²) across whole tissue sections, normalized to CK/SOX10^{+} tumor cell counts.

Flow Cytometry: Single-cell suspensions from PKT pancreatic tumor samples were harvested. Samples were incubated with FcR-blocking reagent (Miltenyi Biotec) and subsequently stained with fluorescently conjugated antibodies listed in the table below. Flow cytometry data was acquired on Cytek Aurora and was processed using FlowJo v.10 software. All flow cytometry procedures, including tissue processing, fluorochrome selection, live/dead sorting, and gating strategies were performed as previously described (3).

**Myeloid Markers**				
**Target**	**Fluorophore**	**Catalog #**	**Clone #**	**Company**
CD45	BUV395	564279	30-F11	BD
CD11b	BUV805	741934	M1/70	BD
F4/80	BV785	123141	BM8	Biolegend
CD86	PerCP-eFlour710	46-0862-82	GL1	ThermoFisher
MHC II	APC-Cy7	116426	AF6-120.1	Biolegend
CD206	BV650	141723	C068C2	Biolegend

LY6G	BUV563	612921	1A8	BD
LY6C	PE-Cy7	128018	HK1.4	Biolegend
Arg-1	APC	17369782	A1exF5	ThermoFisher
CXCR2	FITC	149310	SA044G4	Biolegend
CD162	BV510	563448	2PH1	BD
CD170	PE	155505	S17007L	Biolegend
**Lymphocyte Markers**				
**Target**	**Fluorophore**	**Catalog #**	**Clone #**	**Company**
TCRb	BUV805	748405	H57-597	BD
CD62L	BUV563	741230	MEL-14	BD
CD44	BUV737	612799	IM7	BD
TIGIT	BUV421	142111	1G9	Biolegend
Foxp3	BV421	126419	MF-14	Biolegend
Ly108	Pacific Blue	134608	330-AJ	Biolegend
Tim-3 (havcr2)	BV785	119725	RMT3-23	Biolegend
CD8	BV510	100752	53-6.7	Biolegend
TCF-1	Alexa-Flour 488	IC8224G	812145	Fisher Scientific
KLRG-1	BB700	742199	2F1	ThermoFisher
FoxP3	PE	126404	MF-14	Biolegend
PD-1	PE-Dazzle594	135228	29F.1A12	Biolegend
CD69	PE-Cy5	104510	H1.2F3	Biolegend
CD4	Pe-Cy7	116016	RM4-4	Biolegend
CD39	Alexa-Flour647	143808	Duha59	Biolegend
lineage	Alexa-Flour 700	118240	G8.8	Biolegend
CD127	APC-eFlour 780	47-1271-82	A7R34	ThermoFisher
TOX	APC	130-118-335	REA473	Myltenyi Biotec

Statistics. Descriptive statistics were calculated using Prism 10.4.1 (GraphPad, La Jolla, CA). Continuous variables are reported as mean ± standard deviation. For two group comparisons, a two-tailed Student's t-test was applied. Duration-of-response and survival analyses were performed using Kaplan–Meier estimates with log-rank (Mantel–Cox) tests. A two-sided $p \leq 0.05$ was considered statistically significant.

Study approval. All animal experiments were performed in accordance with the NIH animal use guideline and protocol (21-076) approved by the Institutional Animal Care and Use Committee (IACUC) at the University of Miami. All analyses involving human tissue samples and clinical data were conducted in accordance with institutional guidelines and approved by the University of Miami Institutional Review Board (IRB20200123). All patients enrolled in clinical trials provided written informed consent.

Data availability. Raw data are available in "Supporting data values file". ScRNAseq data from mNadu vs. isotype PKT experiments have been uploaded to GEO database as **GSE310710**. Data values from clinical trial specimens and survival analysis are available upon reasonable request.

Acknowledgements. We wish to acknowledge the following Shared Resources at Sylvester Comprehensive Cancer Center for their able assistance with this project: Biospecimen Shared Resource (BSSR); Flow Cytometry Shared Resource (FSCR); and Oncogenomics Shared Resource (OGSR)

Authors contributions
*contributed equally: Erin M. Dickey; Harper M. Marsh

Experimental conceptualization and methodology performed by: JD, DL, AB, PJH, NM, KVJ

Investigation performed by: EMD, HMM, CRM, HA, KR, MP, A. Adams, A. Allena
Writing of the manuscript performed by: EMD, HMM, JD

Reviewing and editing of the manuscript performed by: HMM, AB, JD

SUPPLEMENTAL FIGURE LEGENDS:

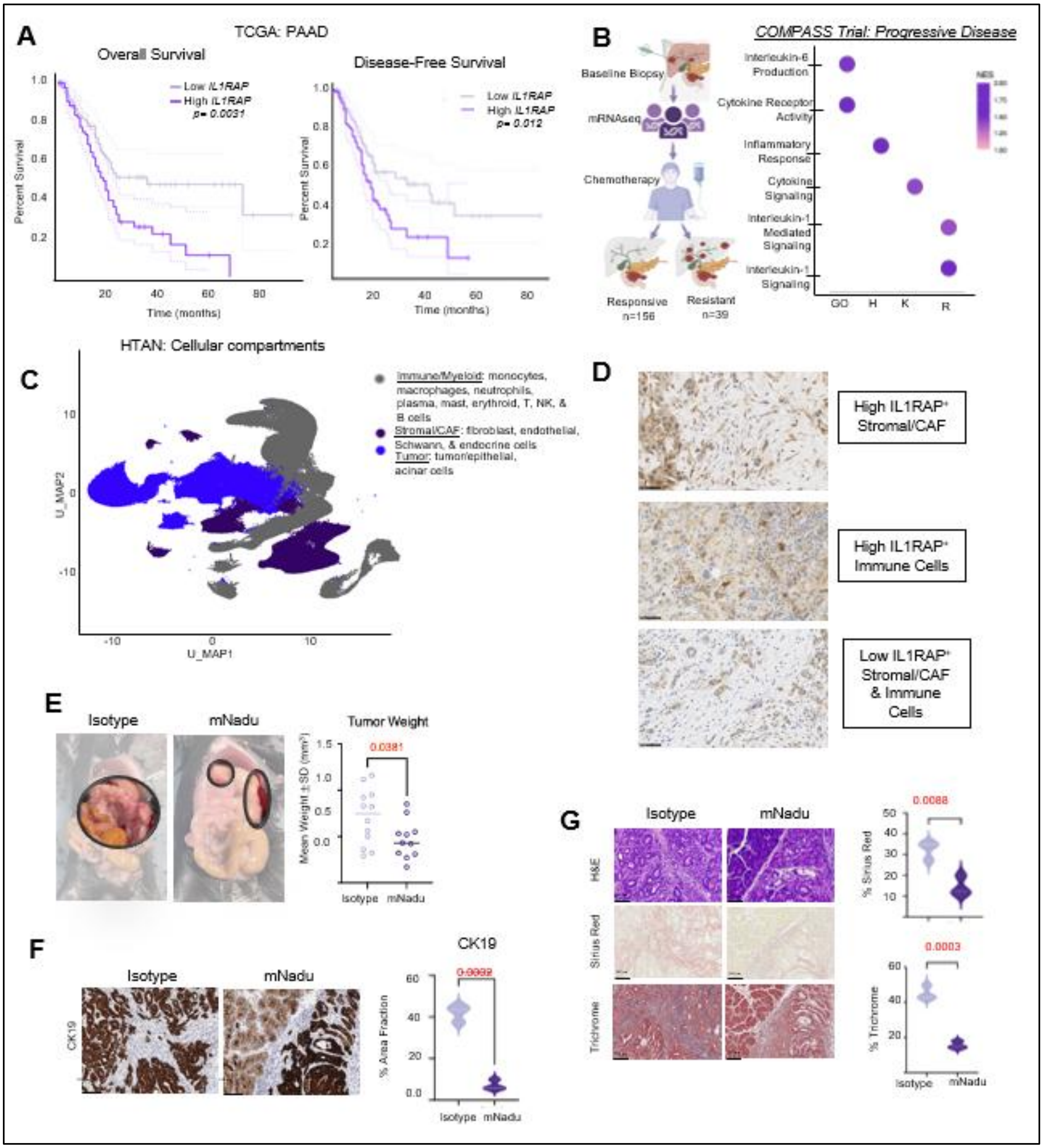

Supplemental Figure S1. (A) Kaplan-Meier survival analysis of overall survival (*left*) and disease-free survival (*right*) in The Cancer Genome Atlas (TCGA) pancreatic adenocarcinoma (PAAD; n=178 samples, log-rank test, two-tailed) samples. “High” and “Low” IL1RAP expression groups (n=89 each) were defined by median dichotomization of *log_2-transformed transcript levels [log_2(TPM + 1)]*; **(B)** Stratification of COMPASS trial samples by chemotherapy-resistant (n=39) versus chemotherapy-responsive (n=156*)*

(*left*) showing enrichment of IL1RAP-associated pathways in resistant tumors (*right*); all FDR q-value<0.05; **(C)** UMAP visualization of 654,997 single cells from the Human Tumor Atlas Network (HTAN) PDAC dataset, grouped by transcriptionally defined major compartments. Tumor/acinar cells are shown in blue; stromal/CAF populations—i.e.,cancer-associated fibroblast (CAF), endothelial, Schwann, and endocrine lineages—are shown in purple; immune/myeloid lineages—i.e., monocytes, macrophages, neutrophils, plasma cells, mast cells, erythroid cells, T-cells, NK cells, and B cells—are shown in dark gray; **(D)** Representative immunohistochemistry (IHC) images of baseline biopsies from patients enrolled in the CANFOUR Phase Ib/IIa trial of nadunolimab (NCT03267316; Van Cutsem *et al*., Clin Cancer Res 2024), showing high IL1RAP expression in stromal/CAF (*top*) or immune (*middle*) compartments, and low IL1RAP expression across both stromal/CAF and immune compartments (*bottom*). Scale bar, 50 μm; **(E)** Representative post-mortem necropsy images highlighting tumor burden in PKT mouse model treated with isotype (*left*) or mNadu (*middle*). Primary tumor weights are shown within the scatter plot (*right*; n=11 mice/group, unpaired t test, two-tailed); **(F)** IHC analysis of CK19 in PKT tumor sections from mice treated with isotype or mNadu (scale bar,100 μm). Violin plots depict quantification of positive fraction $CK19^+$ malignant cell regions using whole slide scanning (n=3 mice/group, unpaired t test, two-tailed); **(G)** H&E, Sirius Red, and Trichrome staining with quantification (n=3/group; scale bar, 100μm)

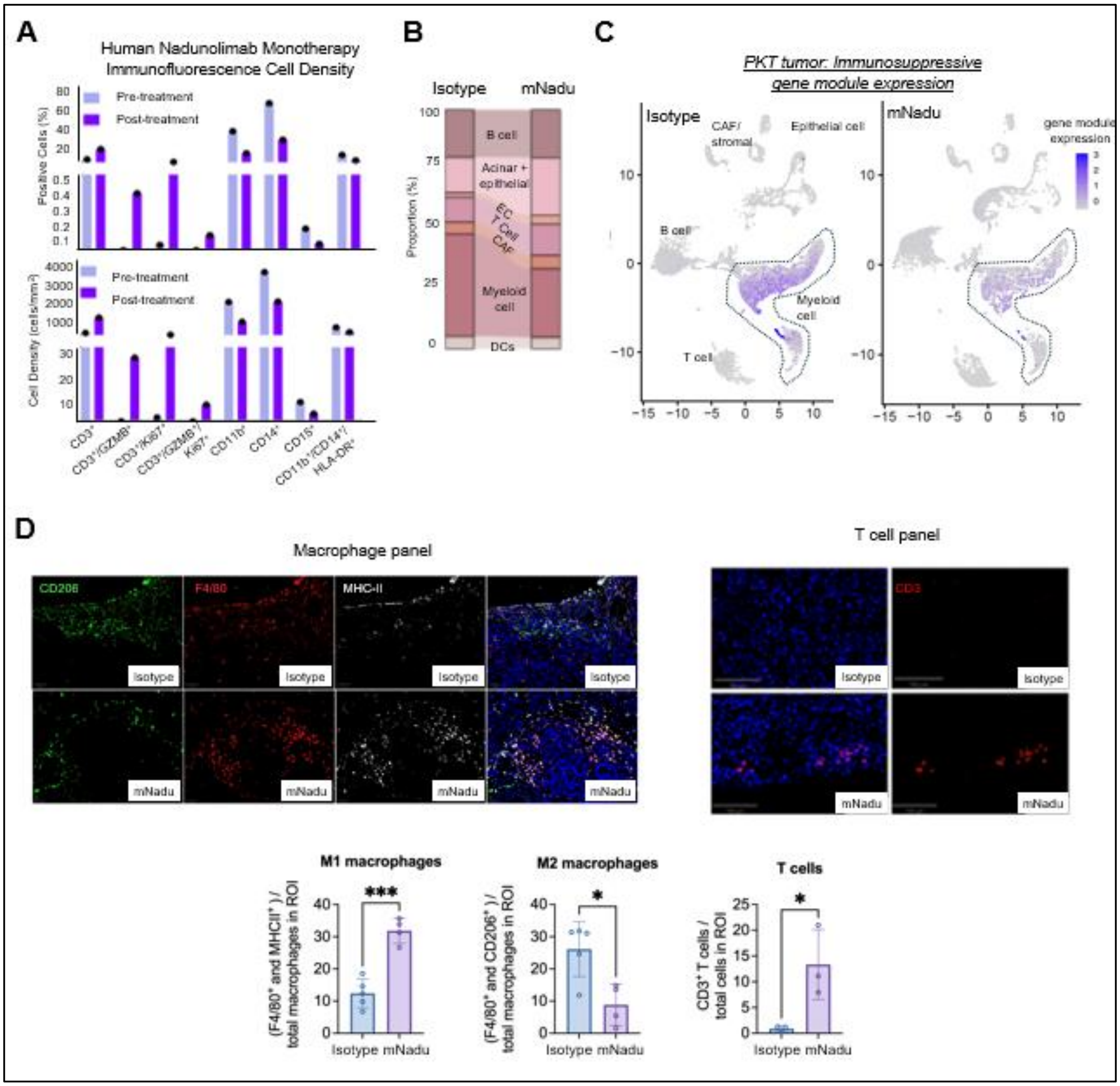

Supplemental Figure S2. (A) Multiplex immunofluorescence (mIF) staining of paired pre- and post-treatment PDAC core biopsies from patients (n=2) treated with nadunolimab monotherapy. Tissue sections were stained using Ultivue InSituPlex® panels—MDSC FixVUE and T-act FixVUE—and quantified for marker-specific positivity expressed as percentage of positive cells and cell density (cells/mm²) across whole tissue sections; **(B)** Alluvial plot depicting cell cluster redistribution across 15,834 single cells from isotype- versus mNadu- treated PKT tumors (n=3/group); **(C)** UMAP projection of 15,834 single cells from PKT tumors treated with isotype control or mNadu (pooled from n=3 mice/cohort), annotated by major cell lineages. Cells are colored by immunosuppressive gene module activity (*Arg1, Cybb, Chil3, Cd177, Camp*), with module scores displayed across myeloid, T-cell, B-cell, epithelial, and stromal/CAF

compartments; **(D)** Representative immunofluorescence staining of tumor sections (n=7/group) from isotype control- and mNadu-treated mice showing macrophage markers (CD206, F4/80, MHC-II; scale bar, 20μM) and T cells (CD3, scale bar, 100μM) (*top*), and quantified in the bar graphs. Cell counts were normalized to total cells per ROI. Each dot represents an individual ROI (n=3) Bars represent mean ± SEM (*bottom*). Statistical significance was determined using unpaired two-tailed *t* tests (* p<0.05, ** p<0.001).

Authors contributions

**contributed equally: Erin M. Dickey (Data curation: Lead; Formal analysis: Lead; Investigation: Lead; Methodology: Lead; Validation: Lead; Writing-original draft: Co-Lead; Writing – review & editing: Supporting); Harper M. Marsh (Data curation: Supporting; Formal analysis: Co-Lead; Investigation: Supporting; Writing- review & editing: Supporting)*

References

1. Knox JJ, Jang GH, Grant RC, et al. Whole genome and transcriptome profiling in advanced pancreatic cancer patients on the COMPASS trial. *Nature Communications*. 2025/07/01 2025;16(1):5919. doi:10.1038/s41467-025-60808-z
2. Cui Zhou D, Jayasinghe RG, Chen S, et al. Spatially restricted drivers and transitional cell populations cooperate with the microenvironment in untreated and chemo-resistant pancreatic cancer. *Nature Genetics*. 2022/09/01 2022;54(9):1390-1405. doi:10.1038/s41588-022-01157-1
3. Datta J, Dai X, Bianchi A, et al. Combined MEK and STAT3 Inhibition Uncovers Stromal Plasticity by Enriching for Cancer-Associated Fibroblasts With Mesenchymal Stem Cell-Like Features to Overcome Immunotherapy Resistance in Pancreatic Cancer. *Gastroenterology*. Dec 2022;163(6):1593-1612. doi:10.1053/j.gastro.2022.07.076

4. Van Cutsem E, Collignon J, Eefsen RL, et al. Efficacy and Safety of the AntiIL1RAP Antibody Nadunolimab (CAN04) in Combination with Gemcitabine and NabPaclitaxel in Patients with Advanced/Metastatic Pancreatic Cancer. *Clinical Cancer Research*. 2024:OF1-OF11. doi:10.1158/1078-0432.Ccr-24-0645
